# Pseudoxanthoma elasticum

**DOI:** 10.1186/s13023-017-0639-8

**Published:** 2017-05-10

**Authors:** Dominique P. Germain

**Affiliations:** Division of Medical Genetics, University of Versailles - Saint Quentin en Yvelines, Paris-Saclay University, 2 avenue de la source de la Bièvre, F-78180 Montigny, France

**Keywords:** Pseudoxanthoma elasticum, Metabolic disease, Ectopic mineralization, Skin, Angioid streak, Choroidal neovascularization, Peripheral arterial disease, *ABCC6*

## Abstract

Pseudoxanthoma elasticum (PXE) is a genetic metabolic disease with autosomal recessive inheritance caused by mutations in the *ABCC6* gene. The lack of functional ABCC6 protein leads to ectopic mineralization that is most apparent in the elastic tissues of the skin, eyes and blood vessels. The clinical prevalence of PXE has been estimated at between 1 per 100,000 and 1 per 25,000, with slight female predominance. The first clinical sign of PXE is almost always small yellow papules on the nape and sides of the neck and in flexural areas. The papules coalesce, and the skin becomes loose and wrinkled. The mid-dermal elastic fibers are short, fragmented, clumped and calcified. Dystrophic calcification of Bruch’s membrane, revealed by angioid streaks, may trigger choroidal neovascularization and, ultimately, loss of central vision and blindness in late-stage disease. Lesions in small and medium-sized artery walls may result in intermittent claudication and peripheral artery disease. Cardiac complications (myocardial infarction, angina pectoris) are thought to be relatively rare but merit thorough investigation. Ischemic strokes have been reported. PXE is a metabolic disease in which circulating levels of an anti-mineralization factor are low. There is good evidence to suggest that the factor is inorganic pyrophosphate (PPi), and that the circulating low levels of PPi and decreased PPi/Pi ratio result from the lack of ATP release by hepatocytes harboring the mutant ABCC6 protein. However, the substrate(s) bound, transported or modulated by the ABCC6 protein remain unknown. More than 300 sequence variants of the *ABCC6* gene have been identified. There is no cure for PXE; the main symptomatic treatments are vascular endothelial growth factor inhibitor therapy (for ophthalmic manifestations), lifestyle, lipid-lowering and dietary measures (for reducing vascular risk factors), and vascular surgery (for severe cardiovascular manifestations). Future treatment options may include gene therapy/editing and pharmacologic chaperone therapy.

## Background

### Disease name and synonyms

Pseudoxanthoma elasticum (PXE); OMIM #264800

Grönblad-Strandberg syndrome

ICD-10: Q82.8; ORPHA #758

### Definition

The term “pseudoxanthoma elasticum” was coined by the French dermatologist Ferdinand-Jean Darier in 1896 [1], by reference to the yellowish tone of skin features (seen in true cases of xanthoma) and the lax aspect of the skin at flexural surfaces. Darier also observed abnormal histological features of the skin. However, skin plaques in what was probably PXE were first described by Rigal in 1881 [2]. The link between retinal angioid streaks and skin features in PXE was reported by Grönblad and by Strandberg in 1929 [3, 4], and PXE is occasionally referred to as Grönblad-Strandberg syndrome. PXE is a genetic disease with autosomal recessive inheritance in which dystrophic calcification (i.e. the abnormal accumulation of calcium/phosphate complexes) leads to cutaneous, ocular, cardiovascular and other manifestations [5, 6]. Most of the published evidence suggests that PXE is a metabolic disease, with decreased plasma pyrophosphate (PPi) levels being one of the strongest candidates for pathophysiology [7–10]. The effects of calcification are most apparent in the elastic tissues in the skin, eyes and blood vessels [11]. The deposits in PXE consist of calcium hydrogen phosphate, calcium hydroxyapatite and, to a lesser extent, iron precipitates [12, 13].

### Epidemiology

The clinical prevalence of PXE has been estimated at between 1 per 100,000 and 1 per 25,000 of the general population, with slight female predominance [14, 15]. However, there are few data on allelic frequencies.

## Clinical description

### Cutaneous manifestations

**Fig. 1:**
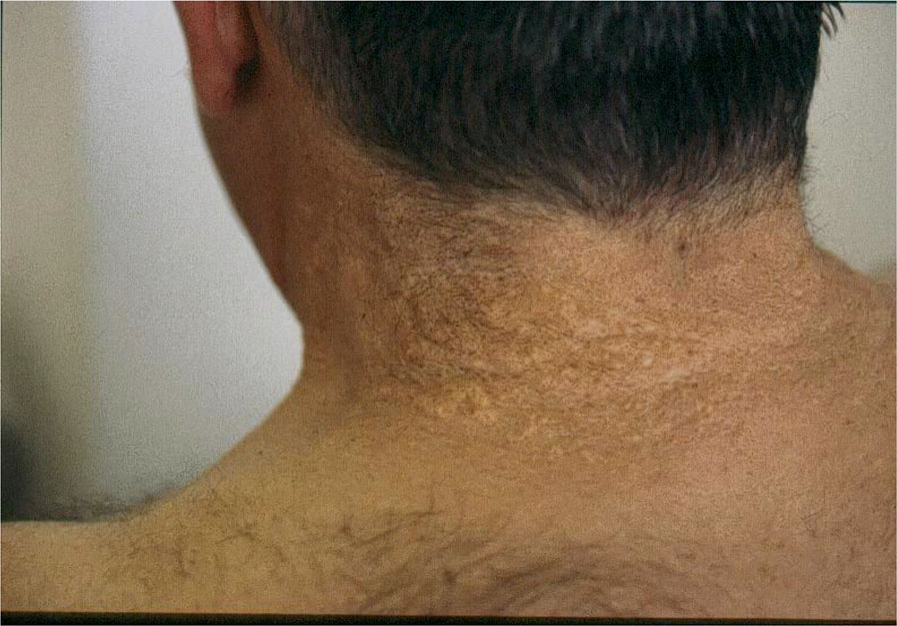
Characteristic cutaneous feature of PXE: yellow papules on the nape of the neck give the skin a peau d’orange aspect

**Fig. 2:**
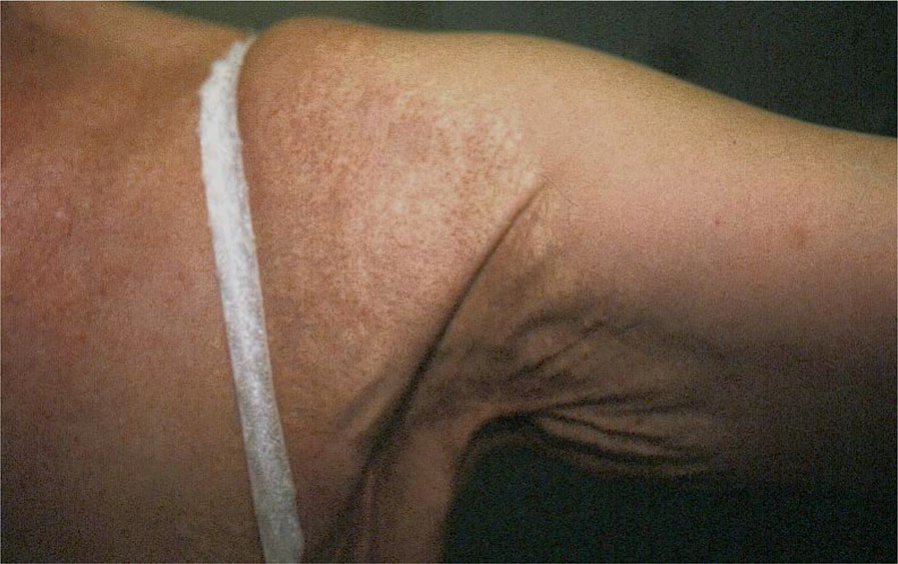
Characteristic advanced cutaneous feature of PXE: involvement of axillary flexural folds

The first clinical sign of PXE, with onset typically in childhood or adolescence [16] tends to be the characteristic skin changes (small yellow papules with diameter of up to 10 mm) on the nape and sides of the neck and in flexural areas (such as the axillae, the antecubital fossae, and periumbilical, inguinal and popliteal areas) [17] (Figs. 1 and 2). The oral, vaginal and rectal mucosae may also be affected. The papules are initially isolated or found in patches but coalesce into reticulated plaques as the disease progresses, giving a cobblestone aspect to the skin. The skin subsequently becomes loose and wrinkled, albeit not to the extent seen in cutis laxa [16]. It has been suggested that the presence of horizontal and oblique mental (chin) creases before the age of 30 years is specific for PXE [18]. Histological features of PXE may be found in the absence of overt skin lesions in patients with angioid streaks and macroscopically normal skin [19]. In rare cases, patients with genetically confirmed PXE may have histologically normal skin [20].

**Fig. 3:**
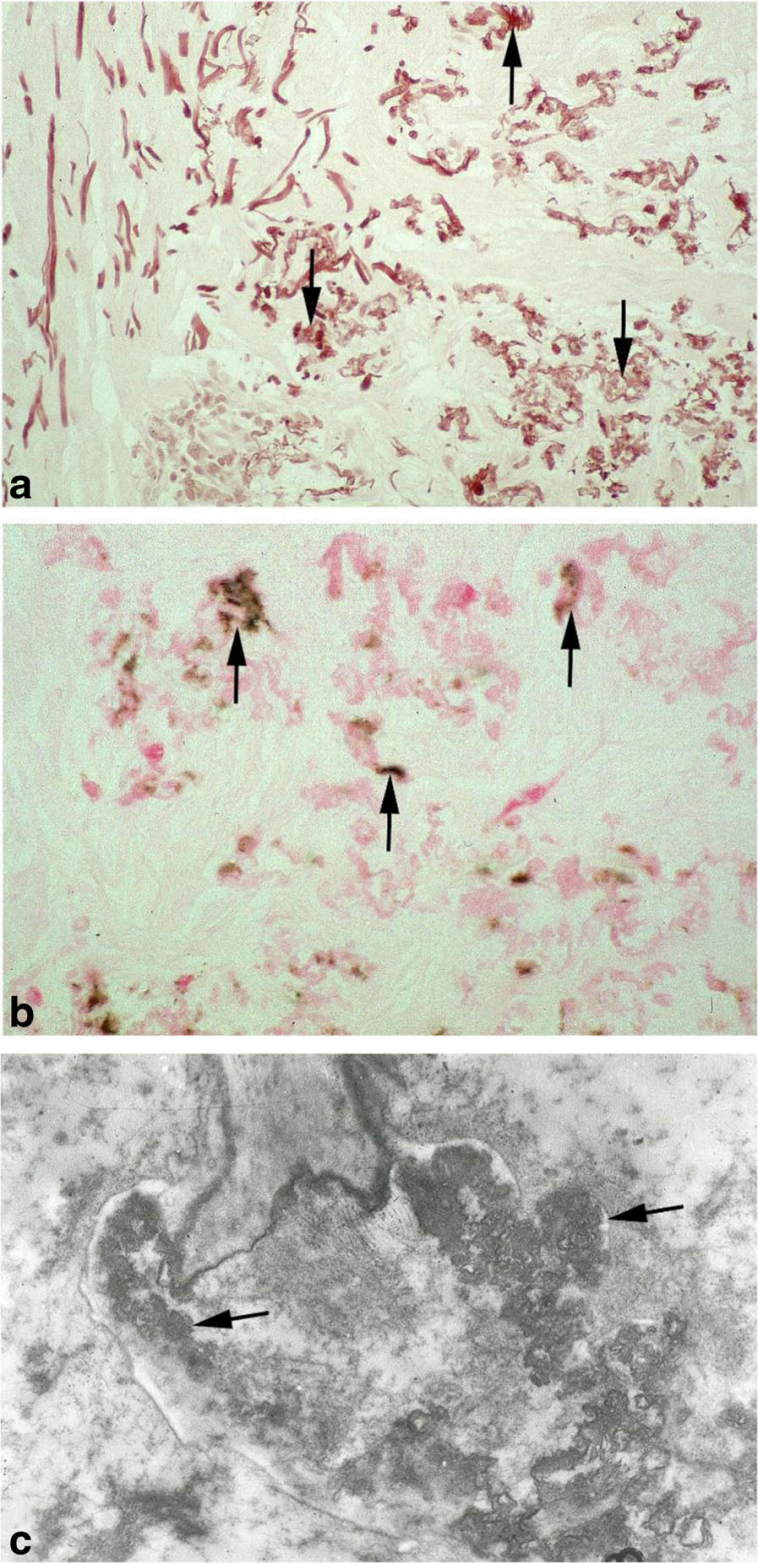
Characteristic histological features of PXE in skin biopsies. **a** Orcein staining: the elastic fibers of the dermis are fragmented and thickened. **b** Von Kossa staining: calcification of elastic fibers. **c** Viewed under the electron microscope, the elastic fibers’ morphology is abnormal

Electron microscopy of the skin reveals bulky, sometimes needle-like mineral deposits that disrupt and break elastic fibers (particularly in the mid-dermis) [13, 21, 22] (Fig. 3). Collagen irregular fibrils have been reported in the skin, myocardium and pericardium [23]. It has been reported that areas of clinically normal skin in PXE patient also contain damaged elastic fibers; it remains to be seen whether this change is an early marker for PXE [21].

### Ophthalmological manifestations

**Fig. 4:**
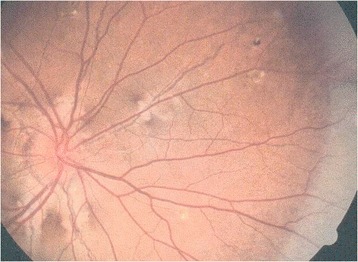
Characteristic ophthalmological feature of PXE: angioid streaks on the fundus

The ophthalmological manifestations of PXE are the most serious, since they can lead to blindness in late-stage disease. The characteristic ocular feature of PXE is the presence of angioid streaks in the retina [24] (Fig. 4). The streaks are variable in color (red/brown/grey) and reflect lesions in Bruch’s membrane - the innermost, elastic layer of the choroid. They can be observed several years after the onset of skin changes. The term “angioid” derives from the streaks’ aspect when viewed in fundoscopy, and these lesions are not vessels *per se*. The angioid streaks may become symptomatic when they approach the fovea of the macula. As the disease progresses, the calcification of Bruch’s membrane may trigger choroidal neovascularization. New subretinal vessels grow through the lesions in Bruch’s membrane, coating the posterior pole of the retina and eventually leading to hemorrhage, scarring, loss of central vision and thus blindness (Fig. 4) if not treated [24–26]. However, angioid streaks are not pathognomic for PXE because they may be present in diseases such as sickle cell disease, thalassemia and, more rarely, Ehlers-Danlos syndrome [24, 27]. It has been reported that angioid streaks are often preceded by drusen-like retinal peau d’orange changes in the temporal part of the macular region [28]. The peau d’orange sign was observed in 96% of patients with skin signs of PXE [16]. “Comet tail”, “punched-out” and “paired wing” lesions have also been described in PXE patients, and it has been suggested that the comet lesions are pathognomonic for PXE [28]. In a study of 107 PXE patients, visual impairment was associated with a major degradation in vision-related quality of life measured with the Impact of Vision Impairment questionnaire [29].

### Vascular and systemic manifestations

Vascular signs (with the exception of claudication) usually become apparent years after the onset of skin and ocular changes. Patients with PXE have an elevated risk of vascular disease because the media and intima of blood vessels (mainly small and medium-sized arteries) are also affected by the dystrophic calcium/phosphate (Pi) mineralization of connective tissue that characterizes this metabolic disease [30]. The primary clinical expression of the arterial wall mineralization is intermittent claudication in both lower and upper limbs and peripheral artery disease [31, 32]. Involvement of the vascular wall (particularly in distal vessels) may lower the success of surgical procedures and should prompt the pre-operative assessment of all candidate vessels [33, 34]. Aneurysms [35, 36], stroke [31, 36], transient ischemic attack [36, 37], stenosis of medium-sized arteries such as the radial and carotid arteries [38, 39], and stenosis of the aorta [39] have also been reported. PXE has been described as a unique monogenic model of peripheral artery disease in which arterial wall remodeling is associated with an abnormally low ankle-brachial index (i.e. the ratio of the systolic blood pressure at the *dorsalis pedis* or posterior tibial artery to the highest systolic blood pressure of the left or right brachial artery), independently of cardiovascular risk factors [32, 40]. In contrast, cardiac complications (myocardial infarction, angina pectoris, etc.) are thought to be relatively rare but, when present, merit thorough investigation [41].

The frequency of ischemic stroke (although not clearly established) appears to be higher than in the general population [42], with a value of 15% in a cohort of 38 PXE patients [31] and 7% in another cohort of 100 patients, giving a relative risk of 3.6 versus the general population [36]. Carotid *rete mirabile* has been reported in association with PXE [37, 42].

It is noteworthy that in a study of 107 PXE patients, it was found that cardiovascular complications of the disease had relatively little impact on health-related quality of life, using the 36-item Short Form Health Survey [29].

Furthermore, it has also been suggested that heterozygous carriers of *ABCC6* mutations (estimated frequency in the general population: up to 1 in 80) have an increased risk of cardiovascular calcification and premature coronary artery disease [15, 43].

Around 15% of PXE patients will experience hemorrhage of the gastrointestinal or urinary tract (especially the stomach), compared with around 0.1% in the general population [16, 25, 44].

Calcification of the kidneys, breasts, pancreas, testicles, liver and spleen has variously been observed in patients with PXE. With the possible exception of the kidneys, this calcification is not thought to have a major clinical impact [28].

PXE may have an impact on some aspects of lung function. In a functional study of 35 PXE patients, 11 had a significantly low carbon monoxide diffusing capacity [45]; this was interpreted as a possible preclinical state for interstitial lung disease.

## Etiology

### Genetics and molecular biology

PXE is a genetic disease with autosomal recessive inheritance. PXE-causing mutations in the *ABCC6* gene on chromosome 16 were discovered in 2000 [46–51]. The *ABCC6* gene consists of 31 exons, coding for a protein of 1503 amino acids (molecular weight: 165 kDa).

In the literature, there are 48 *ABC* (“adenosine triphosphate (ATP) binding cassette”) genes, divided into seven subfamilies (A to G). The *ABCC* subfamily includes 12 genes, including *ABCC6* and *ABCC7* (the latter also being known as *CFTR* – the mutated gene in cystic fibrosis), and a pseudogene (*ABCC13*). For reasons of structural homology, the protein encoded by the *ABCC6* gene has been included in the multidrug resistance protein subfamily, some members of which export organic ions derived from exogenous sources (such as cancer drug metabolites) [52, 53]. Hence, in some older publications, *ABCC6* is referred to as *MRP6*. The ABCC4, ABCC5, ABCC11 and ABCC12 proteins contain two membrane-spanning domains interspersed with two nucleotide-binding domains. The sulf onylurea receptor units SUR1 and SUR2 (encoded by *ABCC8* and *ABCC9*) also have four domains, whereas the ABCC1, ABCC2, ABCC3, ABCC6 and ABCC10 proteins have an additional N-terminus domain. A three-dimensional model of the ABCC6 protein has been proposed by homology with the high-resolution structures of other ABC proteins [54]. However, in the absence of experimental confirmation by X-ray crystallography or high-resolution nuclear magnetic resonance, the accuracy of this model can be questioned.

*ABCC6* gene expression is regulated in a tissue-specific manner [55]. It has been suggested that in addition to the proximal promoter, a primate-specific sequence (+629/+688) in the first intron of the human *ABCC6* gene has a tissue-specific role [56]. The finding that the master regulator hepatocyte nuclear factor 4 α (HNF4α) binds to a highly conserved site (−209/−145) within the promoter may account for the predominant expression of *ABCC6* in the liver [57].

Whether or how endogenous or exogenous substrates are transported by ABCC6 has not been well characterized. Even though ABCC6 had been included in the MRP family by homology, the molecular mechanism by which ABCC6 could transport drugs or their metabolites has not been defined. Hence, ABCC6 is unlikely to be involved in clinical multidrug resistance [53]. According to the results of in vitro experiments with membrane vesicles containing *ABCC6* transfected into Chinese hamster ovary cells, the transfected cells were not notably resistant to etoposide, teniposide, doxorubicin, daunorubicin, actinomycin D or cisplatin [58].

As in any autosomal recessive disease, it is generally accepted that heterozygous carriers of a mutation in one *ABCC6* allele do not develop PXE [59, 60]. However, some heterozygotes seem to display clinical and histopathological features of PXE [61–63]. The observation of abnormally mineralized skin areas in a woman with a p.R1141X mutation in *ABCC6* and a p.V255M mutation in *GGCX* (coding for gamma-glutamyl carboxylase) [64] has prompted the consideration of a *forme fruste* of PXE (OMIM #177850). When considering PXE-like manifestations in heterozygotes, it is possible that an unrecognized mutation affects the second supposedly wild-type allele and thus still corresponds to recessive inheritance [31]. However, as noted above, it has been suggested that heterozygotes for *ABCC6* mutations have an elevated risk of cardiovascular calcification [15].

### Pathophysiology

While the genetic nature of the disease is well recognized, the pathophysiological mechanism of PXE has yet to be fully understood. It has been reported that although ATP secretion from the liver is ABCC6-dependent, ATP itself is not transported by ABCC6. However, the ABCC6-dependent secretion of ATP is the main source of pyrophosphate (PPi) in the circulation [9, 10]. Plasma PPi levels in *Abcc6* ^(−/−)^ mice are around 40% of those found in wild-type mice, and the plasma PPi/Pi ratio is low in PXE patients [9, 10]. Hence, on the basis of experiments in HEK293 cells overexpressing either human or rat ABCC6 and in vivo experiments in *Abcc6* ^(−/−)^ mice, PPi has been proposed as the candidate circulating factor involved in PXE metabolic disease [9, 65].

Although *ABCC6* is expressed primarily in the liver, the kidneys and the intestine in healthy subjects, the damage in PXE patients occurs most obviously at remote sites. Two main hypotheses can be considered. Firstly, the cell-based hypothesis holds that a lack of functional ABCC6 protein at peripheral sites leads to ectopic mineralization [66]. Although cultured fibroblasts taken from PXE patients’ dermis display biochemical and genetic abnormalities [66, 67], the cell-based hypothesis is weakened by the fact that *ABCC6* mRNA is expressed at only low to moderate levels in tissues outside the liver in healthy controls [47] [68].

The second, predominant paradigm for PXE is that of a systemic, metabolic disease in which the lack of production or release of one or more circulating factors from the liver (where *ABCC6* is usually most strongly expressed) leads to ectopic mineralization. One variant of this metabolic hypothesis holds that the circulating factor usually suppresses or controls mineralization. Hence, in the absence of functional ABCC6 protein, the lack of these circulating factors leads to systemic, dystrophic mineralization throughout the body, including the skin, eyes and arteries. In a striking experimental proof of the metabolic disease hypothesis in the *Abcc6*-deficient (*Abcc6* ^*(−/−*)^) mouse model, the absence of functional abcc6 protein in the mutant was complemented by parabiotic heterogenetic pairing (surgical joining of the circulation with that of a wild-type mouse). The pairing stopped the connective tissue mineralization in the *abcc6* ^(−/−)^ mouse – supposedly through the reintroduction of one or more critical anti-mineralization factors present in the wild-type mouse blood in sufficient quantity [69].

As mentioned above, PPi has been convincingly proposed as the candidate anti-mineralization circulating factor in PXE [9, 65]. High Pi levels have been mentioned as a calcification factor in PXE, on the basis of dietary supplementation experiments in the *abcc6* ^*(−/−)*^ mouse model [70]. However, PXE patients have a normal parathyroid hormone status, and a placebo-controlled clinical trial of an orally administered sevelamer hydrochloride phosphate binder failed to demonstrate a significant effect on elastic fiber calcification and clinical lesions in PXE [71]. However, the latter results may have been biased by the presence of magnesium stearate in the excipient. If Pi does have a role in PXE pathophysiology, it has been proposed it would rather be exerted through the decreased PPi/Pi ratio [9, 10].

Other molecules with a suggested role in PXE are the anti-mineralization proteins matrix Gla-protein (MGP) and fetuin-A, with a suggested link to chronic kidney disease (CKD). Serum levels of MGP and fetuin-A are moderately low in PXE patients [72] and abnormally low in patients with CKD [73]. The MGP knockout mouse shows spontaneous calcification of arteries and cartilage [74]. Interestingly, a murine model of CKD displayed low levels of Abcc6 protein but normal *Abcc6* mRNA levels – suggesting a post-transcriptional or post-translational deficiency [75].

On the basis of animal model experiments, it has also been hypothesized that low vitamin K export from the liver would decrease the gamma-carboxylation of anti-mineralization proteins [76, 77]. Furthermore, MGP is not carboxylated in the elastic fibers of PXE patients [78], and PXE-like calcification of elastic fibers is observed in patients with mutation in the GGCX gene [78]. However, the failure of supplementation trials in murine models of PXE weakens the vitamin K hypothesis [79–81].

Adenosine is another candidate for the circulating factor in PXE, in view of the similarities between PXE and the disease known as “arterial calcification due to deficiency of CD73” (ACDC, in which extracellular adenosine monophosphate cannot be converted to adenosine) [82, 83]. Indeed, patients with ACDC and CD73-deficient mice develop dystrophic calcification, [84, 85]. However, this hypothesis is weakened by the lack of adenosine transport by ABCC6 in in vitro experiments [86].

It has also been suggested that oxidative stress is a pathophysiologic factor in PXE because (i) some PXE patients display biochemical signs of oxidative stress [87], (ii) some patients with β-thalassemia or sickle cell anemia – both conditions in which systemic free radical levels are elevated - can display PXE-like manifestations [88–91], and (iii) oxidative stress inhibits expression of *ABCC6* gene in human cell lines. In the mouse, there is one report suggesting that abcc6 protein localizes to the mitochondria-associated membrane [92]. However, studies of frozen mouse and human liver sections and primary hepatocytes have clearly demonstrated that the main cellular location of ABCC6 protein is the basolateral plasma membrane [93].

Lastly, on the basis of microarray gene expression analyses of wild-type, *Abcc6* deficient and *Abcc6*-transgenic mice [94, 95], it was postulated that the failure of mutant 6 to export one or more substrates from hepatocytes induces changes in the regulation and expression of genes encoding or modulating systemic anti-mineralization factors (the “hepatic intoxication” hypothesis). However, the differences in gene expression were small and were not significant after correction for multiple testing [94], and the changes in the liver’s metabolic profile did not appear to be reflected in the plasma profile [95]. Furthermore, liver function in general is not perturbed in patients in patients with PXE.

Most of the detailed experimental data on the pathophysiology of PXE comes from *Abcc6*-deficient models in the zebrafish [77, 96, 97] and in the mouse [98–102]. The zebrafish model is a useful tool for testing potential therapies, such as premature termination codon read-through [103]. However, this model’s experimental value is limited by the fact that the fetus dies about a week after fertilization [97]. In the mouse, all the *Abcc6* ^−/−^ models develop dystrophic mineralization, with deposits in skin, retina and arteries that resembles the features of PXE in humans. For example, arterial calcium accumulation is 1.5- to 2-fold higher in *Abcc6* ^*−/−*^ knock-out mice than in wild-type mice [104]. A study of *Abcc6*-deficient mice highlighted the activation of the bone morphogenic protein 2 (BMP2)-SMAD-RUNX2 signaling pathway – a critical mediator of vascular calcification [105].

Genotype-phenotype correlations are generally weak [61]. It has been suggested that the nonsense mutation p. Arg1141* might predispose patients to cardiovascular disease, independently of hyperlipidemia [43, 62, 63, 106, 107] and that the *ABCC6* p. Arg1268Gln polymorphism [50] is associated with the early onset of the disease’s characteristic angioid streaks [108, 109]. *ABCC6* mutations have also been occasionally linked to a lethal disorder known as generalized arterial calcification of infancy (GACI; OMIM 173335) associated with mutations in the *ENPP1* gene coding for the ectonucleotide pyrophosphatase/phosphodiesterase-1 regulator of bone mineralization [110]. Death occurs in utero or in the first few months of life. Mutations in *ENPP1* on chromosome 6q23 have been found in the majority of patients with GACI [111].

## Diagnosis

### Clinical criteria

There are no widely accepted and applied international guidelines for the clinical and genetic diagnosis of PXE. Historically (and notably before the discovery of the *ABCC6* gene’s causal role in PXE), patients were screened for three major criteria and two minor criteria [112]. The three major criteria were (i) characteristic skin involvement with yellow cobblestone lesions in flexural locations, (ii) characteristic histopathologic features of the lesional skin, with elastic tissue or von Kossa stains, and (iii) characteristic ocular disease, with angioid streaks, peau d’orange lesions or maculopathy in adults older than 20 years of age. The two minor criteria were characteristic histopathologic features of non-lesional skin and a history of PXE in first-degree relatives. However, this historical classification does not always fit well with molecular data on *ABCC6* [60].

A new classification was proposed in 2010 (Table 1) [28]. It comprises a semi-standardized work-up: (i) examination of the skin by a dermatologist or specialist physician familiar with PXE, (ii) hematoxylin–eosin, Verhoeff–van Gieson (elastin) and von Kossa (calcium) staining of a skin biopsy from an affected lesion (Fig. 3) or, if not applicable, a biopsy from the lateral side of the neck, (iii) fundoscopy of the posterior pole of both eyes by an experienced ophthalmologist (checking for peau d’orange, angioid streaks, macular degeneration, comets, and wing signs), and optional fluorescein or indocyanine green angiography and fundus autofluorescence (for angioid streaks) [28]. In practice, the presence of characteristic yellow cobblestone skin lesions alone will usually prompt screening for *ABCC6* mutations.

**Table 1:** Revised diagnostic criteria for PXE (adapted from [28])

Major diagnostic criteria
1. Skin a. Yellowish papules and/or plaques on the lateral side of the neck and/or flexural areas of the body; or b. Increase of morphologically altered elastin with fragmentation, clumping and calcification of elastic fibers in a skin biopsy taken from clinically affected skin
2. Eye a. Peau d’orange of the retina; or b. One or more angioid streaks (ASs), each at least as long as one disk diameter. When in doubt, fluorescein or indocyanine green angiography of the fundus is needed for confirmation.
3. Genetics a. A pathogenic mutation of both alleles of the ABCC6 gene; or b. A first-degree relative (parent, sib, child) who meets independently the diagnostic criteria for definitive PXE
Minor diagnostic criteria
1. Eye a. One AS shorter than one disk diameter; or b. One or more ‘comets’ in the retina; or c. One or more ‘wing signs’ in the retina
2. Genetics a. A pathogenic mutation of one allele of the *ABCC6* gene
Requirements for the diagnosis of PXE
a. Definitive diagnosis The presence of two (or more) major criteria not belonging to the same (skin, eye, genetic) category
b. Probable diagnosis The presence of two major eye or two major skin criteria, or The presence of one major criterion and one or more minor criteria not belonging to the same category as the major criterion
c. Possible diagnosis The presence of a single major criterion, or The presence of one or more minor criteria

Sickle cell anemia, beta-thalassemia, and PXE-like phenotype with cutis laxa and multiple coagulation factor deficiency should be excluded, if mutational analysis of ABCC6 is negative or not available. Signs and symptoms in PXE may arise with increasing age. If a patient is <30 years a probable or possible diagnosis of PXE should be considered provisional and dermatologic and ophthalmologic examinations should be repeated after 5 years

### Laboratory diagnosis

#### Biochemical diagnosis

There are no specific or generally informative biochemical assays for PXE. Hemoglobin profiling and vitamin-K-dependent coagulation factor assays may be used to rule out sickle cell disease, beta thalassemia and multiple coagulation factor deficiency [28].

#### Molecular biology

As mentioned above, patients will be screened for *ABCC6* mutations unless the clinical findings are unambiguous. More than 300 unique DNA sequence variants of the *ABCC6* gene (mostly missense mutations) have been identified to date [https://www.ncbi.nlm.nih.gov/clinvar/?term=*ABCC6*[gene]]. Around 90% of patients with clinical PXE will have a mutation in both alleles.

The mutational profile varies from one ethnic group to another [113]. For example, the p.Arg1141* (p.R1141X) mutation is common in European populations [113], less common in Northern American populations [114] and was absent in a group of 22 Chinese patients (in whom 15 previously unreported mutations were detected) [115]. The del23-29 mutation is common in northern Europe and the northern Mediterranean region, whereas the p.Gly1321Ser mutation is prevalent in North America but rare in Europe [114]. The p.Arg1138Trp missense mutation may be a marker for French descent (since it is found in France and in French-speaking Canada), whereas the 2542delG frameshift mutation occurs predominantly in Japanese patients [113]. In contrast, the prevalence of p.Gln378* and p.Arg1339Cys mutations appear to be similar worldwide, suggesting recurrent mutational events. Overall, disease-causing missense mutations appear to be concentrated at domain–domain interfaces, with a 4.25-fold higher mutation rate [54]. Copy number variations in the two *ABCC6* pseudogenes *ABCC6Ψ1* and *ABCC6Ψ2* [116, 117] have been found to be more common in PXE patients than in controls, although the clinical significance of this, if any, is unclear [118, 119].

Non-disease-causing polymorphisms have been identified; interestingly, an individual who was homozygous for an *ABCC6* p.Arg1268Gln polymorphism did not have symptoms of PXE, and the Gln1268 (Q1268) allele had a frequency of 0.19 in healthy controls [50].

#### Histology

##### Light microscopy

Elastin is stained with Verhoeff–van Gieson reagent, and calcium deposits are revealed with Von Kossa staining [11, 17] (Fig. 3). The mid-dermal elastic fibers are short, fragmented, clumped and calcified. These characteristics are strongly suggestive of PXE but not pathognomic. Elastic fiber clumping and calcification are only present in clinically affected skin in mutated *ABCC6* homozygotes or compound heterozygotes [28]. Splitting, thickening, coiling, calcification and flower-like deformation of skin collagen fibers are observed in some but not all PXE patients [16] and so are not thought to be clinically relevant.

As in the skin, histochemical assessment of Bruch’s membrane also reveals calcium deposits [12]. Similarly, elastic fibers become mineralized and disrupted in blood vessel walls, the myocardium and the pericardium [23]. Arterial vessels are most strongly affected, although fragmentation of elastic fibers in the vena cava has also been reported [23].

## Differential diagnosis

### Dermatological and connective tissue diseases

Intense solar elastosis of the nape of the neck in elderly people can mimic the macroscopic aspect of PXE skin features [120]. PXE-like macroscopic skin lesions are also observed after chronic D-penicillamine therapy [121] and in “acquired PXE” (perforating calcific elastosis, a non-inherited skin disease mainly affecting the peri-umbilical region in multiparous women) [122]. Some of the features of PXE can arise in rare dermatological disease such as late-onset focal dermal elastosis [123], papillary dermal elastolysis [124], mid-dermal elastolysis [125] and PXE-like skin manifestations with retinitis pigmentosa [78]. As mentioned above, angioid streaks can very occasionally be observed in Ehlers-Danlos syndrome. All these differential diagnoses can be ruled out by genetic testing for *ABCC6* mutations.

### β-thalassemia and sickle cell anemia

As mentioned above, skin manifestations resembling those seen in PXE and (in some cases) angioid streaks have been observed in individuals with β-thalassemia and sickle cell disease who clearly lack *ABCC6* gene mutations [89, 90]. Hence, angioid streaks are not pathognomonic for PXE. Thalassemic patients with PXE-like skin lesions also manifest PXE-like vessel alterations that progress with time [126]. Interesting, progressive, liver-specific down-regulation of *abcc6* was found in a murine model of β-thalassemia [127].

### Body skin hyperlaxity due to vitamin K dependent coagulation factor deficiency

Body skin hyperlaxity due to vitamin K-dependent coagulation factor deficiency is an autosomal recessive disorder caused by mutations in either the *GGCX* or *VKORC1* gene [128, 129]. Although the disorder is not associated with *ABCC6* gene mutations, patients may display skin manifestations similar to those observed in PXE and cutis laxa [130]. In PXE with diffuse skin folds, screening for *GGCX* mutations can be considered. However, the disease progression is quite different, with the development of leathery lesions [131].

## Management

### Management of cutaneous manifestations

Although aesthetic concerns can prompt some patients to seek treatment for nuchal and axillary symptoms of the disease [132–134], surgery for these non-life-threatening symptoms should be implemented with caution [17].

The suggested role of oxidative stress in PXE has prompted an *ad hoc* attempt at antioxidant therapy with daily doses of tocopherol acetate and ascorbic acid in one patient [63]. The skin lesions had regressed at 12 months but had started to progress again at 18 months. Furthermore, administration of an antioxidant diet in the *Abcc6* ^−/−^ mouse model had no effect on mineralization [135].

### Management of ophthalmologic manifestations

Intravitreal treatment with vascular endothelial growth factor (VEGF) inhibitors (such as bevacizumab) has rapidly become an effective treatment for stopping choroidal neovascularization – often the most critical symptom of PXE [136–138]. Accordingly, physical treatments such as photodynamic therapy have become less extensively used. Contact sports should be avoided, due to the risk of retinal hemorrhage.

### Management of vascular and systemic manifestations

The current treatment approach for slowing or limiting the cardiovascular manifestations of PXE is based on the reduction of cardiovascular risk factors through lifestyle changes (smoking cessation, weight loss, daily walking, moderate physical exercise, etc.). In terms of drug treatment, a survey of 1,747 patients with PXE (reported on in a study of atorvastatin administration in a murine model of PXE) suggested that a third were taking or had taken cholesterol-lowering agents [139]. Acetylsalicylic acid is typically contraindicated in PXE, due to the increased likelihood of bleeding from a diseased retinal neovasculature [140]. In particular, patients with gastrointestinal hemorrhage should avoid nonsteroidal anti-inflammatory drugs and antiplatelet agents [15]. However, this risk must be balanced against the potential benefits in the prevention of thrombophilia.

In the event of arterial stenosis, standard surgical bypass or percutaneous angioplasty can be performed [31, 32]. Weakness of the vascular wall (particularly in distal vessels) may alter the choice of vessels for surgical grafts and should prompt the pre-operative assessment of all candidate vessels. For example, use of the saphenous vein may be preferable to the highly patent internal mammary artery, which can also be affected, for coronary bypass [33, 34].

The various pathophysiological hypotheses for PXE (involving putative circulating pro- or anti-mineralization factors) have prompted researchers to test the effects of dietary supplementation in animal models and humans. Magnesium supplementation improved some disease indicators in the *Abcc6* ^*(−/−)*^ mouse [141, 142]. Twice-daily magnesium oxide supplementation has been tested in PXE patients in a 2-year clinical trial (ClinicalTrials.gov NCT01525875). However, the results had not been published at the time of writing. It has also been suggested that a high calcium intake in early life correlates with severity of PXE, although it is not known whether a low-calcium diet in infancy would be feasible with a view to restricting ectopic mineralization. In experiments with *Abcc6* ^*−/−*^ and *Enpp1* ^*asj*^ mice, the administration of high oral doses or lower subcutaneous doses of bisphosphonates or etidronate prevented ectopic mineralization [143, 144].

Lastly, it has been postulated that protein conformation modifiers may allow synthesis of a functional, full-length ABCC6 protein. In in vitro experiments with polarized MDCKII cells [145], wild-type ABCC6 protein localized to the basolateral plasma membrane. The drug sodium 4-phenylbutyrate (approved as a treatment for urea cycle disorders) [146] restored the plasma membrane localization of four “mistargeted” *ABCC6* mutants (p.Arg1114Pro, p.Ser1121Trp, p.Gln1347His, p.Arg1314Trp) in vitro and in vivo in the mouse liver [145, 147]. Encouragingly, treatment with sodium 4-phenylbutyrate also reduced dystrophic calcification in the *Abcc6* ^*−/−*^ mouse [148]. However, the small number of mutants tested means that this approach must be further characterized and studied.

### Gene therapy

PXE is a candidate for gene therapy. Given that mutant *ABCC6* heterozygotes have few or no features of PXE, the presence of one healthy allele or moderate expression should be enough to relieve the symptoms of the disease. Since *ABCC6* is most strongly expressed in the healthy liver, targeting a transgene to this organ is logical. Novel technologies and delivery options for liver-directed gene therapy are being developed [149, 150]. In the rodent, efficient gene transfer to the liver can be conveniently obtained by tail vein injection of viral and non-viral vector systems [149, 151]. Plasmid-based gene therapy has been tested in the *Abcc6* ^−/−^ murine model of PXE [152]. A cDNA encoding human *ABCC6* was subcloned into a non-viral, liver-specific expression vector carrying the mouse albumin promoter and a fetoprotein enhancer. The vector was delivered by a single tail vein injection of 3-month-old *Abcc6* ^−/−^ mice. Functional human ABCC6 protein was transiently expressed in 13% of the animal’s hepatocytes, on average. Expression was associated with significantly less intense calcification 3 to 7 days after induced cardiac cryoinjury [152].

However, several shortcomings of the gene therapy approach will need to be overcome [151]. As required for all gene therapies, it will be essential to check that delivery of an *ABCC6* transgene is safe and does not induce severe immune reactions or insertional oncogenesis [153].

## Genetic counselling

PXE is transmitted according to a Mendelian autosomal recessive inheritance, with a 25% risk of recurrence in siblings.

Although calcification of the placenta and low birth weight have been reported, the risk of pregnancy is not elevated for both the fetus and the mother, and there is no reason to contraindicate pregnancy. Since inheritance is autosomal recessive, children conceived by a PXE patient and a non-affected individual will not be affected – except in cases of endogamy or genetic isolates in which pseudodominance has been reported [154].

## Prenatal diagnosis

In theory, the discovery of causal mutations in *ABCC6* has rendered prenatal testing and preimplantation genetic diagnosis possible [49]. However, given that PXE is not life-threatening, the ethical justification for prenatal diagnosis is subject to debate.

## Unresolved questions and perspectives

- The recently proposed “metabolic hypothesis” for PXE [7–9] has opened up some interesting opportunities for mechanistic and therapeutic research. An appropriate PPi/Pi ratio is critical for prevention of ectopic mineralization under homeostatic conditions and the most prominent candidate for the dystrophic calcification observed in PXE is decreased PPi/Pi ratio. Hence, there is a clear need for robust, double-blind, placebo-controlled clinical trials of dietary treatments, anti-mineralization agents, anti-osteoclastic drugs, vitamin K, etidronate, anti-oxidants and pharmacologic chaperones [145, 147, 155, 156] in PXE patients, with support from disease advocacy organizations [157, 158].
- Genotype-phenotype correlations must be better defined. Next-generation sequencing, bioinformatics and the various "omics" technologies are now being used to study regulation and expression of *ABCC6*, and to search for possible disease modifier genes [66, 84, 154, 159, 160].
- While heterozygous carriers of autosomal recessive diseases are typically considered to be healthy, several publications have emphasized the potential association between heterozygosity for the p.R1141X *ABCC6* mutation and a variety of more common conditions, such as coronary artery disease [43]. Further research on digenism and/or putative modifier genes would be of value [154, 159, 160].
- Liver-directed gene therapy/editing may become a treatment option in the future if stable, liver-specific expression is ensured, *ABCC6*-modified hepatocytes have a growth advantage, and any potential safety concerns have been addressed.

## Conclusions

PXE is now a well-characterized, autosomal recessive, metabolic, genetic disease of ectopic mineralization that affects the skin, eye and blood vessels. Although not life-threatening, PXE is associated with a risk of blindness, decreased quality of life and peripheral vascular compromise. There is no cure for PXE and patients should be monitored on a regular basis (clinical examinations, exploration of the vascular tree with MR angiography and ultrasound, fundus examination of the posterior pole of both eyes). Behavioral and lifestyle factors include moderate exercise and the avoidance of trauma to the eyes. If quality of life is significantly impaired by skin manifestations, plastic surgery can be considered. Some precautions should be taken prior to vascular surgery. Although the exact pathophysiological mechanisms underlying the metabolic disease have yet to be identified, the suggested role of PPi as the circulating anti-mineralization factor should open up opportunities for the clinical development and validation of disease-modifying treatments.

## Abbreviations

ACDC: Arterial calcification due to deficiency of CD73
BMP2: Bone morphogenic protein 2
CKD: Chronic kidney disease
GACI: Generalized arterial calcification of infancy
GGCX: Gamma-glutamyl carboxylase
HNF4α: Hepatocyte nuclear factor 4 alpha
MGP: Matrix Gla-protein
MRP: Multidrug resistance protein
PPi: Pyrophosphate
PXE: Pseudoxanthoma elasticum
VEGF: Vascular endothelia growth factor
